# Perceptions of Ghanaian traditional health practitioners, primary health care workers, service users and caregivers regarding collaboration for mental health care

**DOI:** 10.1186/s12913-021-06313-7

**Published:** 2021-04-23

**Authors:** Solomon Nyame, Edward Adiibokah, Yasmin Mohammed, Victor C. Doku, Caleb Othieno, Benjamin Harris, Oye Gureje, Seedat Soraya, John Appiah-Poku

**Affiliations:** 1 Kintampo Health Research Centre, Box 200, Kintampo, Bono East Region Ghana; 2 Population Council, Accra, Ghana; 3 Kwame Nkrumah University of Science and Technology, Kumasi, Ghana; 4 Kings College London, London, UK; 5 University of Nairobi, Nairobi, Kenya; 6 University of Liberia, Monrovia, Liberia; 7 University of Ibadan, Ibadan, Nigeria; 8 Stellenbosch University, Stellenbosch, South Africa

**Keywords:** Traditional health practitioners, Collaboration, Primary health care, Complementary healthcare

## Abstract

**Background:**

In low- and middle-income countries, the paucity of conventional health services means that many people with mental health problems rely on traditional health practitioners (THPs). This paper examines the possibility of forging partnerships at the Primary Health Care (PHC) level in two geopolitical regions of Ghana, to maximize the benefits to both health systems.

**Methods:**

The study was a qualitative cross-sectional survey. Eight (8) focus group discussions (FGDs) were conducted between February and April 2014. The views of THPs, PHC providers, service users (i.e. patients) and their caregivers, on the perceived benefits, barriers and facilitators of forging partnerships were examined. A thematic framework approach was employed for analysis.

**Results:**

The study revealed that underlying the widespread approval of forging partnerships, there were mutual undertones of suspicion. While PHC providers were mainly concerned that THPs may incur harms to service users (e.g., through delays in care pathways and human rights abuses), service users and their caregivers highlighted the failure of conventional medical care to meet their healthcare needs. There are practical challenges to these collaborations, including the lack of options to adequately deal with human rights issues such as some patients being chained and exposed to the vagaries of the weather at THPs. There is also the issue of the frequent shortage of psychotropic medication at PHCs.

**Conclusion:**

Addressing these barriers could enhance partnerships. There is also a need to educate all providers, which should include sessions clarifying the potential value of such partnerships.

## Background

The role played by traditional or indigenous health practitioners in healthcare service delivery in low- and middle- income countries (LMICs) is widely acknowledged [1–5]. Traditional medicine is “the total of knowledge, skills and practice based on the theories, beliefs and experiences indigenous to different cultures that are used to maintain health as well as to prevent, diagnose, improve or treat physical and mental illness” [6]. Estimation suggests that about 80% of the African population rely on THPs [7]. A complex interplay of factors drives the continued use of THPs, including prevailing belief systems and practices (which are associated with the attribution of spiritual causes to illness), widespread distribution of THPs, weak conventional healthcare systems, and socio-economic and cultural factors [8–14]. In most LMICs, such as Ghana, the use of THPs is not a barrier to the use of conventional medicine and patients using THPs continue to make use of conventional medicine [1, 15, 16] underpinning the need to utilize best practices from both systems to ensure optimal healthcare delivery [2, 5].

Since the 1970s, there have been calls for partnership between THPs and mainstream healthcare (WHO, 2008). Most African countries have formulated national traditional medicine policies and regulatory frameworks for the development of traditional medicine [17]. The Mental Health Act, enacted in Ghana, encourages partnerships between the two health systems [18]. The emphasis has since shifted from the need for partnership between both healing systems in LMICs to the practical issues of implementation required for successful partnerships [19].

The partnership that is expected to be between complementary alternative providers of mental health service (CAPs) and conventional or orthodox providers, represented by Primary Health Care Providers (PHCPs). In the Ghanaian setting, CAPs are made up of two main groups: traditional healers and faith healers. Traditional healers may fall into subgroups of herbalists (those who use plant products for medicinal purposes) or diviners (those who claim to gain insight for healing by occultic or ritual processes). herbs and divination are used as treatment modalities is not uncommon. Faith healers will often belong to either the Christian or Islamic faith. These healers rely on prayers and religious rituals, including divination and sacrifices to provide healing.

Most attempts at partnerships between CAPs with conventional medicine in sub-Saharan Africa have not been very successful due to multifaceted challenges [19–22]. Three main reasons advanced in the literature for the failure of such partnership efforts: i) “inadequate understanding of the context” whereby practical operational issues are not properly examined before implementation; ii) “uninformed skepticism and mistrust”, namely that some opponents hold very strong opposition to partnerships without recourse to the available evidence; and iii) “uncritical enthusiasm” of stakeholders involved, whereby some proponents promote partnerships without noting the potential impeding factors [23–26]. It is important for the success of any form of partnership to examine critically the views of the stakeholders involved against the background of contextual factors that promote or impede such partnerships [27]. Indeed, Konadu 2007, in his study among the Bono of the Brong Ahafo Region of Ghana, cautioned against the uncritical “integration” of THPs into conventional medical care as this could lead to predictable failure [28].

There has been a fair amount of research on mental health and CAPs in Ghana [29, 30]. However, very few studies have comprehensively contrasted the views of all key stakeholders at PHC level and examined the possibility of partnerships between THPs and PHC providers. Ae-Ngibise et al., 2010, highlights the widespread use of traditional and faith healers in Ghana and notes the need, and potential, for collaboration [24]. Arias et al., 2016, employing 50 open-ended, semi-structured interviews with Christian healers and staff at nine Christian prayer camps in Ghana, and with staff within Ghana’s three public psychiatric hospitals, explored whether the prayer camp and the biomedical beliefs and practices of staff provided sufficient common ground to enable cooperative relationships. The study concluded that prayer camp staff were interested in collaboration with biomedical mental health care providers, particularly if the partnership could provide technical support, introduce medications in the prayer camp and address key shortcomings in their infrastructure and conditions of hygienic [31].

This paper aims to examine the views of three stakeholders namely, THPs (Traditional and Faith Healers), Primary Health Care Providers, Service Users (Patients) and their Caregivers, regarding the possibility of fostering partnerships between THPs and PHCPs in Ghana to maximize benefits to both health systems. This study constituted one of the formative studies conducted by the Partnerships for Mental Health Development in sub-Saharan Africa (PaM-D) project. The PaM-D project had the broader aim of providing evidence to support innovative approaches to integrating THPs into PHC in the context of relatively few available physicians and specialist mental health professionals in sub-Saharan Africa. This study is a National Institute of Mental Health funded project between five African Countries (Nigeria, Ghana, South Africa, Kenya and Liberia). The overall aim of the PaM-D project was to ensure effective delivery of evidence-based interventions for psychosis through a process of collaborative care, training, support and supervision [32].

## Method

The study took place in two of the ten political, administrative regions of Ghana, Ashanti and Brong-Ahafo which are both in the middle belt of the country. These two regions together account for about 30% of Ghana’s total population. Despite the population density in these regions, none of the three (3) psychiatric hospitals in Ghana is located in these regions. However, there are psychiatric units at the Komfo-Anokye Teaching Hospital, Kumasi and the Brong-Ahafo Regional Hospital, Sunyani. There is a total of 590 PHCs in both Ashanti (364) and Brong Ahafo regions (226). A good number of them have community psychiatric nurses (CPNs) under a task-shifting arrangement and THPs serve as the de facto mental health service providers in these conventional psychiatric service environments.

The Kwame Nkrumah University of Science and Technology Committee on Human Research Publication and Ethics (CHRPE) approved the study protocol. Informed consent was obtained from all participants who either signed or provided thumbprint consent. For participants who were illiterate (patients and caregivers), witnesses who were literate were relied on to explain the study information sheets to them. These illiterate participants had the opportunity to ask questions and to have them addressed before their consent (in the form of thumbprints) was taken. All data were anonymized prior to analysis.

### Data collection and participant selection

Focus group discussions (FGDs) among purposively selected healthcare service providers (faith healers, traditional healers and primary healthcare workers) and mentally ill persons and their caregivers who had recently sought care from THPs formed the main data source. The selection was based on THPs and PHCs who at the time of the discussion had patients with mental disorders. Two persons were selected from each participating PHCs and THPs. None of the participants declined to participate in the discussions. The purpose and objective of the study were explained to the participants before the start of the discussions. The FGDs were moderated by SN and EA, who were trained at the Masters degree level and with over ten years experience in the conduct of qualitative data collection. Fieldwork was conducted between February 2014 and April 2014. At that time of fieldwork, both EA and SN were the Project Coordinators of the study, and they were both males. Based on the objectives of the study, a discussion guide was designed for all the study sites, and this was discussed across the sites to address content- and context-specific issues. Discussions were audio recorded. All discussions were conducted in Twi and translated into English, after transcription. Back-translation checks were conducted by an independent bilingual English-Twi speaker to validate the translation. However, FGDs with PHC providers were conducted in English and transcribed verbatim. In all, eight FGDs were conducted across the two regions, with each discussion group composed of 7 to 12 participants. These were made up of 23 PHC workers, 28 THPs (14 faith healers, and 14 traditional healers) and 16 service users and their caregivers (8 service users and 8 caregivers). FGDs were employed as they allow for examination of group level phenomena, structure, process and outcome. For the health care provider discussions, the two groups were made up of providers who treat mental illness at the community level. This included Community Mental Health Officers, Community Psychiatric Nurses and Clinical Psychiatric Officers (see Table 1). Also, while the discussions with caregivers and users were conducted on the premises of the healer, these discussions were conducted in a private setting away from the healer (to ensure openness and freedom of expression). The other discussions were conducted in a neutral setting. The average time for each FGD discussion was an hour. The study team conducted field visits to the study settings. Before the discussions, the moderators (SN and EA) and participants introduced themselves. The moderator summarized the aims and procedures for the study and participants had the chance to ask questions.

**Table 1:** Participant selection for the Focus Group Discussions (FGDs)

Total of 8 FGDs: Primary Health Care Providers (2), Traditional Health Care Practitioners (4) and Users and Caregivers (2)	
Focus Group Discussions (FGDs)	Justification/Selection criteria
**Primary Healthcare Providers -Two (2) FGD*s*** ***Ashanti Region (11 participants)*** - 3 females, 8 males - Categories of PHC 1 Psychiatrist 8 Community Psychiatric Nurses 1 Medical Assistant 1 Midwife ***Brong- Ahafo Region (12 participants)*** - 7 females, 5 males - Categories of PHC 1 Psychiatrist 10 Community Psychiatric Nurses 1 Medical Assistant	FGDs with PHC Providers included all categories of PHC workers who deal with mental illness (Psychiatrists^a^, Community Psychiatric Nurses, Medical Assistants and Midwifes). Efforts were also made to ensure both male and female PHC workers were adequately represented.
**Traditional Health Practitioners (THPs) -Four (4) FGD*s*** ***Ashanti Region (14 participants)*** - Categories of THPs **1 Traditional Healer FGD** 1 female and 6 males **1 Faith Healer FGD** 2 females and 5 males ***Brong Ahafo Region (14 participants)*** **1 Traditional Healers** 1 female and 6 males **One (1) Faith Healers** 3 females and 4 males	Selection of THPs included all of the broad categories of THPs, including faith-based healers (Muslim and Christian Healers), and traditional healers (mix of spiritualists [fetish priests] and herbalists). A greater number of FGDs were, therefore, conducted for this group*.* These were purposively selected as they were currently managing patients with psychosis.
**Users and Caregivers –Two (2) FGD*s*** ***Ashanti Region (8 participants)*** 5 females and 3 males Categories of Caregivers & Users Patients (4) Caregivers (4) ***Brong Ahafo Region (8 participants)*** 5 females and 3 males Categories of Caregivers & Users Patients (4) Caregivers (4)	Selection included users of varying sex and age. Since the team wanted caregivers to corroborate what patients said we included both patients and their respective caregivers in the same group discussion. They were purposively selected based on the patient’s ability to provide informed consent as well as a psychiatrist confirmation of the person’s ability to participate in the group discussions.

^a^Each PHC FGD included a Psychiatrist invited from the two regional hospitals to enrich the discussions

#### Characteristics of discussants

Table 2 describes the characteristics of the discussants for the FGDs conducted. A further description by age, sex and status.

**Table 2:** Characteristics of discussants

***Patients and caregivers***	
**Characteristic**	**Frequency n(%)**
**Sex**	
Female	11 (68.8)
Male	5 (31.2)
**Age Group**	
Less than 20	1 (6.3)
20–30	6 (37.5)
31–40	4 (25)
41+	5 (31.2)
**Status**	
Caregiver	8 (50)
Patient	8 (50)
***Complementary providers of mental health care***	
**Characteristic**	**Frequency n(%)**
**Sex**	
Female	6 (20.7)
Male	23 (79.3)
**Age Group**	
Less than 20	–
20–30	–
31–40	13 (44.8)
41+	16 (55.2)
**Status**	
Traditional Healer	14 (50.0)
Faith Healer	14 (50.0)
***Primary Healthcare providers***	
**Characteristic**	**Frequency n(%)**
**Sex**	
Female	10 (43.5)
Male	13 (56.5)
**Age Group**	
Less than 20	–
20–30	11 (47.8)
31–40	6 (26.1)
41+	6 (26.1)
**Role**	
Community Mental Health Officer	10 (43.5)
Community Psychiatric Nurse	11 (47.8)
Other^a^	2 (8.7)

^a^Other include Psychiatrist, Psychologist, Midwife and Physician Assistants

### Data analysis

**Fig. 1:**
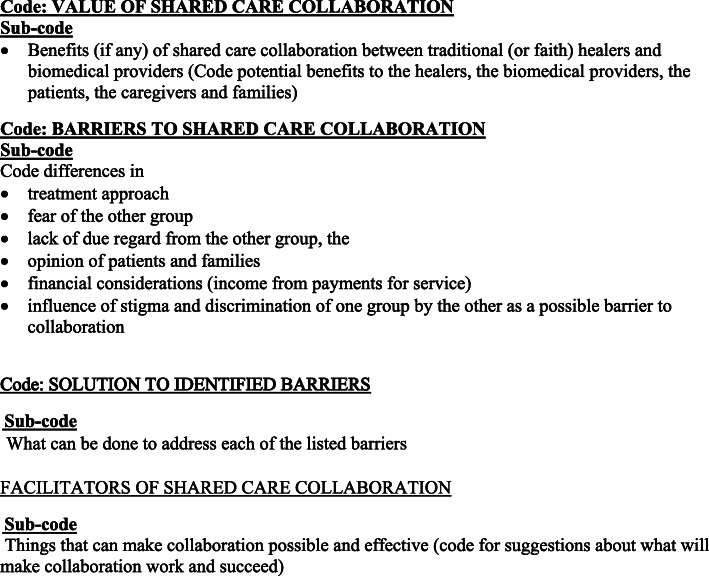
Themes for analysis

A thematic framework approach was adopted for the analysis [33]. Various themes and sub-themes were collectively agreed upon by the investigators across the study sites, following the study objectives. Figure 1 shows the various themes used for the analysis.

Analysis was undertaken at the Ghana site based on the coding frame developed for all sites. The first and second author coded the transcriptions on the basis of a pre-determined coding frame (based on the objective of the study). After that, specific themes emerging from the transcripts were added to the framework during the analysis, and transcripts were coded accordingly. MAXQDA version 11 (VERBI Software, Berlin, Germany) was used for the analysis. The first and second author undertook training in the use of MAXQDA software for the analysis. Three groups of stakeholders were compared: i) THPs composed of faith healers and traditional healers; ii) PHC workers; and iii) service users and their caregivers. Four broad themes were considered: i) opinions on the value of collaboration; ii) barriers to collaboration; iii) factors that could improve the collaboration; and iv) facilitators of shared care collaboration. Coding involved both inductive and deductive approaches allowing emerging themes to be incorporated. Each transcript was coded twice by EA and SN (the first and second author) to establish inter-coder reliability. All differences were discussed under the supervision of JAP until we achieved consensus.

## Results

This study provides qualitative insights into the possibility of forging workable partnerships between THPs and PHC workers from the perspectives of key partners involved in such partnerships (THPs, PHC workers, service users and their caregivers). Four broad themes were considered: i) opinions on the value of this collaboration; ii) barriers to the collaboration; iii) factors that could improve such collaboration; and iv) facilitators of shared care collaboration.

### Benefits of partnerships

The results of our study indicate that across stakeholder groups there was widespread support for collaboration. In the stakeholder FGDs, participants were asked if they thought collaboration was beneficial. There was general support for collaboration among stakeholders. They indicated that such collaboration made it possible to utilize the beneficial aspects of both healing systems. Service users and their caregivers were generally in support of partnerships. Many of the THPs in the FGDs also supported partnerships. PHC workers were of the opinion that partnerships offer the opportunity for THPs to deal with emergencies and with physical ailments. Surprisingly a few PHC workers endorsed the referral of service users to THPs. Previous studies have indicated that PHC workers were against referrals to the THPs and would only support referrals from THPs to PHCs [34]. In one of the FGDs, a PHC service provider suggested that THPs may contribute to getting patients with mental illness well again.

*… it is the cure we are looking for, but they don't get completely healed in our system, so if we partner with them (THPs) and the patient can get well to do his daily activities then I think it is a big benefit* **(Respondent 6, FGD, PHC Workers**)*.*

### Barriers to partnerships

In terms of barriers to partnership, the fear of disrespect from the other group and undue criticism were the two main barriers that came up strongly in the FGDs. THPs were of the view that PHC workers did not respect their form of treatment. Some PHC workers insisted that any form of partnership would first require an investigation of herbal preparations, since such herbal preparations could be medically harmful to service users. PHC workers also feared that patients would suffer human rights abuses and undue delays with THPs; this point of view was particularly supported by the psychiatrists in the FGDs. Service users and caregivers who had been referred to PHCs were also concerned about criticism from and castigation by PHC practitioners. In the FGDs, service users and caregivers who had visited THPs first before visiting PHCs, complained of castigation and abuse at the hands of PHC workers for delays in treatment seeking**.**

*Should you sustain any injury and therefore decide to go to hospital for treatment, a doctor will question you about why you chose to stay at a prayer camp to be treated for mental illness instead of visiting a hospital because his belief is different* **(Respondent 3, FGD***,* **Service Users***)*

Logistical arrangements were another key barrier. This came out in the FGDs with both the PHC sand THPs. PHC workers noted that the frequent stock-outs of psychotropic drugs could hinder partnerships by making them lose “trust” in service users and THPs as frequent shortages of drugs do not bode well for recovery, while THPs indicated that the lack of space to house patients made compliance with human rights challenging and also hampered such collaborations.

*Let us also talk about the drug aspect, if you are in partnership with someone and a patient is referred to you for treatment and you give drugs to the patient for about two to three months and his condition does not improve, and there are frequent shortages of drugs, it will be a big challenge because the other healer will ask us what we are doing in the hospital since we claim to be the best*

(Respondent 2, FGD, **PHC Workers**)*.*

*I even have a patient tied to a tree at my place. But there is nothing I can do since the rooms are occupied and I can't ask them to take him home. So, it is a big problem we are facing and we need support.*

**(Respondent 1, FGD, Traditional Healers***).*

Some service users and their caregivers endorsed THPs and preferred an arrangement whereby PHC workers come in only to confirm that patients have been healed by THPs.

*The doctors can receive us after we have been healed by the Pastor to check on our whole system which will not be so difficult to do because by then the madness would have been gone, and there would be no spiritual interference with the findings of the doctor*

**(Respondent 4, FGD, Patient and Caregivers)**

It was particularly striking that while some PHC practitioners were concerned that the involvement of THPs may be harmful to service users (e.g., through delays in care pathways and human rights abuses), some service users and their caregivers highlighted the failure of conventional medical care to meet their health needs, a situation that reaffirmed their unflinching belief in THPs.

### Facilitators of partnerships

Discussants of the FGDs were asked about factors that would facilitate partnerships between the two healing systems. The majority of THPs expressed the view that PHC workers should respect their opinions and their healing practices. Some THPs reported that PHC workers would demonstrate real commitment to partnerships if they referred patients to them. They insisted that referrals should be bi-directional. PHC workers identified the need to promote mutual respect and recognition of practitioners of both healing systems. They also admitted that there was a need for greater efforts at rapprochement from the PHC workers. A call was made for stakeholders, especially PHCs, to move beyond rhetoric and demonstrate practical commitment to partnership, by refraining from levelling undue criticism on service users who first seek care from THPs. In one of the PHC FGDs, there was a call for a platform that engaged both systems and ensured effective collaboration.

*… . The one thing that will make the partnerships more effective is that there should be review meetings so that we meet once or twice in a year. Then all these people (referring to THPs and services users and their caregivers) will come and we will all review the work we have done in the year or in previous months and we can then look at it to see where there are deficits and consider the way forward.* **(Respondent 2, FGD, PHC Workers***).*

Service users and caregivers also recommended that dealing with the logistical needs of THPs could facilitate partnerships and help to address human rights issues.

*Some of the mad people are even chained to trees. When the sun scorches or it rains, they are left alone.... So, people who are touched by the bishop's (referring to faith healer) unrelenting efforts can help by building him additional rooms for some of the mad people to sleep in*

**(Respondent 6, FGD, Patient and Caregivers)**

## Discussion

The findings of this study are consistent with other studies conducted on the African continent. Although there was widespread support for partnerships across all stakeholder groups, the concerns voiced must be taken seriously if mutually beneficial partnerships are to be forged. As was observed by Ae-Ngibise et al.; 2010, “underneath the dominant rhetoric of support for alliances was widespread pessimism and disapproval”. An equally interesting finding in this study was the mutual suspicion voiced by participants.

To be successful, any form of partnership has to deal with the concerns of users and caregivers about the criticism from PHC workers when they are referred to them. As Incayawar, 2009 aptly puts it, undue critical attitudes ultimately discourage service users and their caregivers from benefiting from the advantages of both systems [2]. This finding, coupled with the admission by PHC workers that the conventional system does not guarantee full recovery from mental illness, gives credence to earlier findings that challenge the often-held notion that the choice of treatment sector by service users and their caregivers is subjectively driven by beliefs about disease causation [29, 35].

It is not surprising that one of the main practical barriers identified by THPs was the lack of options to adequately deal with human rights issues, such as chaining (patients are sometimes chained to immovable objects to ensure that they do not leave the facility). This is sometimes done in the open and chained patients are exposed to the vagaries of the weather. This finding is consistent with a study by Read et al., 2009 who identified the lack of options for humane treatment for service users, both under family care and THPs [24, 31, 36].

### Limitations of the study

Service users and their caregivers mainly comprised those who were currently accessing THP services. All discussions were conducted in the THP setting and this may have affected discussion responses as participants may not have felt comfortable criticizing their treatment providers. This was, to some extent, mitigated by ensuring that discussions were conducted in private. All participants were assured of the confidentiality of their responses. Another limitation of the study was that it focussed on service users with psychosis only, although several of the findings may also be generalisable to persons with other forms of mental illness. Also, although FGDs are effective in eliciting group level responses, they do not lend themselves to personal revelations and may not be a suitable forum to address sensitive issues.

## Conclusion

Practical challenges to collaboration, including the lack of means to deal adequately with human rights issues, such as chaining and exposure to the vagaries of the weather at THPs, and the frequent shortage of psychotropic medication at PHCs, ought to be addressed. Addressing these barriers could enhance partnerships. Any attempts at these collaborations will need to address mutual suspicions openly, with a view to removing unfounded suspicions and finding consensus where genuine concerns exist so that common ground for cooperation can be found. There is also the need to educate PHC providers on the acceptance of service users and their prior treatment-seeking decisions, which could include sessions on clarifying the value of partnerships.

Ghana has experienced healthcare policy and system reforms in recent decades resulting in medical pluralism in the country that calls for more effective partnerships between healing systems [37]. However, in order to ensure that patients are able to maximize the benefits of both healing systems, some of the practical implementation issues confronting such partnerships must be investigated and dealt with. As proposed by Kaboru (2006) “both systems need to acknowledge and accept the limits of their expertise” [38]. This calls for both systems to be offered mutual learning opportunities and a platform to engage in “reflexivity” and to negotiate a partnership [39].

Further studies on the factors that promote or impede successful partnerships between the two healing systems are needed. It is important to examine the lived experiences of users and caregivers seeking care from PHC and THP settings to inform future partnerships.

## Data Availability

The data will be made available on reasonable request to the corresponding author.

## Abbreviations

CPNs: Community Psychiatry Nurses
FGDs: Focus Group Discussions
LMICs: Low-income and Middle-income Countries
PHC: Primary health care
PHCPs: Primary Health Care Providers
THP: Traditional health practitioners
WHO: World Health Organization
